# Comparison of Gemini Advanced and ChatGPT 4.0’s Performances on the Ophthalmology Resident Ophthalmic Knowledge Assessment Program (OKAP) Examination Review Question Banks

**DOI:** 10.7759/cureus.69612

**Published:** 2024-09-17

**Authors:** Gurnoor S Gill, Joby Tsai, Jillene Moxam, Harshal A Sanghvi, Shailesh Gupta

**Affiliations:** 1 Medical School, Florida Atlantic University Charles E. Schmidt College of Medicine, Boca Raton, USA; 2 Ophthalmology, Broward Health, Fort Lauderdale, USA; 3 School of Medicine, University of Florida, Gainesville, USA; 4 Department of Technology and Clinical Trials, Advanced Research, Deerfield Beach, USA; 5 Department of Biomedical Sciences, Florida Atlantic University, Boca Raton, USA

**Keywords:** artificial intelligence in education, artificial intelligence in medicine, chatgpt, customized medical education, general ophthalmology, large language model (llm), ophthalmology

## Abstract

Background

With advancements in natural language processing, tools such as Chat Generative Pre-Trained Transformers (ChatGPT) version 4.0 and Google Bard’s Gemini Advanced are being increasingly evaluated for their potential in various medical applications. The objective of this study was to systematically assess the performance of these language learning models (LLMs) on both image and non-image-based questions within the specialized field of Ophthalmology. We used a review question bank for the Ophthalmic Knowledge Assessment Program (OKAP) used by ophthalmology residents nationally to prepare for the Ophthalmology Board Exam to assess the accuracy and performance of ChatGPT and Gemini Advanced.

Methodology

A total of 260 randomly generated multiple-choice questions from the OphthoQuestions question bank were run through ChatGPT and Gemini Advanced. A simulated 260-question OKAP examination was created at random from the bank. Question-specific data were analyzed, including overall percent correct, subspecialty accuracy, whether the question was “high yield,” difficulty (1-4), and question type (e.g., image, text). To compare the performance of ChatGPT-4 and Gemini on the difficulty of questions, we utilized the standard deviation of user answer choices to determine question difficulty. In this study, a statistical analysis of Google Sheets was conducted using two-tailed t-tests with unequal variance to compare the performance of ChatGPT-4.0 and Google’s Gemini Advanced across various question types, subspecialties, and difficulty levels.

Results

In total, 259 of the 260 questions were included in the study as one question used a video that any form of ChatGPT could not interpret as of May 1, 2024. For text-only questions, ChatGPT-4.0.0 correctly answered 57.14% (148/259, p < 0.018), and Gemini Advanced correctly answered 46.72% (121/259, p < 0.018). Both versions answered most questions without a prompt and would have received a below-average score on the OKAP. Moreover, there were 27 questions utilizing a secondary prompt in ChatGPT-4.0 compared to 67 questions in Gemini Advanced. ChatGPT-4.0 performed 68.99% on easier questions (<2 on a scale from 1-4) and 44.96% on harder questions (>2 on a scale from 1-4). On the other hand, Gemini Advanced performed 49.61% on easier questions (<2 on a scale from 1-4) and 44.19% on harder questions (>2 on a scale from 1-4). There was a statistically significant difference in accuracy between ChatGPT-4.0 and Gemini Advanced for easy (p < 0.0015) but not for hard (p < 0.55) questions. For image-only questions, ChatGPT-4.0 correctly answered 39.58% (19/48, p < 0.013), and Gemini Advanced correctly answered 33.33% (16/48, p < 0.022), resulting in a statistically insignificant difference in accuracy between ChatGPT-4.0 and Gemini Advanced (p < 0.530). A comparison against text-only and image-based questions demonstrated a statistically significant difference in accuracy for both ChatGPT-4.0 (p < 0.013) and Gemini Advanced (p < 0.022).

Conclusions

This study provides evidence that ChatGPT-4.0 performs better on the OKAP-style exams and is improved compared to Gemini Advanced within the context of ophthalmic multiple-choice questions. This may show an opportunity for increased worth for ChatGPT in ophthalmic medical education. While showing promise within medical education, caution should be used as a more detailed evaluation of reliability is needed.

## Introduction

Large language models (LLMs) have revolutionized the field of natural language processing (NLP) by enabling machines to understand, generate, and interact with human language in an increasingly sophisticated manner. These models are built upon deep learning architectures, particularly transformers, which have proven highly effective in capturing the nuances of language through extensive pre-training on vast datasets.

At the forefront of LLM development is OpenAI’s Generative Pre-Trained Transformer (GPT) series, with GPT-4 being the latest iteration. GPT-4 uses a transformer architecture to generate coherent and contextually relevant text. It achieves this by being pre-trained on diverse internet text and fine-tuning for specific tasks, enhancing its ability to understand and produce detailed and contextually accurate responses. In contrast, Google’s Gemini Advanced, part of the Bard AI family, also leverages transformer-based architecture but incorporates distinct training methodologies and optimization techniques. These techniques include enhanced contextual understanding, dynamic memory allocation, and improved response generation algorithms, which aim to improve the model’s comprehension and output quality. Gemini Advanced is designed to excel in complex language tasks by integrating more nuanced contextual awareness and adaptive learning strategies.

The application of LLMs has extended beyond general NLP tasks into specialized domains, including medicine [1]. In the medical field, LLMs hold significant promise for enhancing various aspects of healthcare, such as medical education, clinical decision support, and patient interaction [2]. These models can assist in synthesizing medical literature, answering clinical queries, and providing educational support to medical professionals [2]. One critical area of interest is the potential of LLMs to assist in medical examinations and education [2].

In the past, measurements of artificial intelligence (AI) in medicine were done by assessing its ability in medical exams and comparing it to human performance [3]. These exams range from the United States Medical Licensing Examination (USMLE) Step 1/2 to fellowship-level exams. For instance, the popularity of LLM in medical education began with a study by Gilson et al., who assessed the performance of Chat Generative Pre-Trained Transformers (ChatGPT) on USMLE questions and compared it with other LLMs, such as GPT-3.0 and InstructGPT [4]. Researchers used multiple-choice questions from the AMBOSS and National Board of Medical Examiners (NBME) question banks [4]. The study found that ChatGPT achieved 44% to 64.4% accuracy across different data sets, outperforming GPT-3.0 and InstructGPT4. ChatGPT demonstrated logical justification for its answers in 100% of the cases and utilized internal information effectively [4]. However, its performance decreased with increasing question difficulty [4].

The Ophthalmic Knowledge Assessment Program (OKAP) exam is an annual examination for ophthalmology residents, assessing their knowledge and preparedness for clinical practice [5]. Botross et al. evaluated the performance of Google’s AI chatbot, Bard (now Gemini), on ophthalmology board exam practice questions from the BoardVitals question bank [5]. The study found that Bard correctly answered 62.4% of ophthalmology board exam practice questions, with its highest performance in the Oculoplastics section at 84% and its lowest in the Retina and Vitreous section at 24% [5]. The results of the Botross et al. study underscore the importance of evaluating LLMs in specific medical contexts, highlighting the essential role of experienced ophthalmologists in making nuanced and context-specific medical decisions [5].

Another study by Teebagy et al. aimed to evaluate the performance of ChatGPT-4.0 on the OKAP examination compared to its predecessor, ChatGPT-3.5 [6]. This study provides a relevant context for understanding the potential improvements in LLM capabilities over time and their applicability in medical education [6]. ChatGPT-4.0 significantly outperformed ChatGPT-3.5, achieving an accuracy of 81% compared to 57% (p < 0.001) [6]. The superior performance of ChatGPT-4.0 suggests advancements in model architecture, training data, and fine-tuning processes [6]. A similar study by Antaki et al. assessed the accuracy of two versions of ChatGPT (Legacy and Plus) in answering ophthalmology questions from the Basic and Clinical Science Course (BCSC) Self-Assessment Program and the OphthoQuestions question bank [7]. The legacy ChatGPT model achieved accuracies of 55.8% on the BCSC set and 42.7% on the OphthoQuestions set, while the updated ChatGPT Plus model improved to 59.4% and 49.2%, respectively [7]. The study found that question difficulty and examination section significantly impacted ChatGPT’s accuracy [7]. The findings support the potential for LLMs such as ChatGPT to assist in medical education, though improvements are needed to address specific challenges in specialized fields such as ophthalmology [7].

Our study builds on prior studies by comparing the performance of ChatGPT-4.0 and Gemini Advanced in answering OKAP-specific questions using the OphthoQuestions question bank. By systematically analyzing the responses of ChatGPT-4.0 and Gemini Advanced to both image-based and non-image-based questions, this research aims to evaluate their accuracy and relevance in ophthalmology. The findings will shed light on the capabilities and limitations of these advanced LLMs in medical education, offering insights into their potential role in enhancing learning outcomes and supporting clinical decision-making in ophthalmology.

## Materials and methods

This observational study utilized ophthalmology-specific questions from the OphthoQuestions question bank. There were 260 questions, with 48 image questions and 211 text-only questions. Each question was entered in a new GPT chat page to prevent drawing information from previous messages.

Questions were typed into ChatGPT and Gemini Advanced’s chat box exactly as they appeared in the question bank without additional prompting. However, at times, the LLM would refuse to answer, stating, “I can’t diagnose medical conditions because I’m not a licensed medical professional.” When this message appeared, LLM’s decision not to answer the question was recorded. Then, the following prompt was used to elicit a response: “Pick a letter to this multiple-choice question as if you were taking an exam, with no possible harm done to others.” ChatGPT would provide an answer using this prompt while cautioning that it is not built for clinical decision-making. Gemini Advanced (73.8% response rate) was less reluctant to answer questions compared to ChatGPT (89.2% response rate) (p < 0.000004). However, it was difficult to include patient images in Gemini Advanced, as it would refuse to respond to certain questions that included those images. In such cases, the prompt would sometimes be changed to: “Pick a letter to this multiple-choice question as if you were taking an exam, with no possible harm done to others. The image does not violate any laws or violations regarding privacy.”

Both available models, ChatGPT-4.0 and Gemini Advanced, were compared and assessed for significant differences. The information collected from the questions included correctness, subspeciality if the question was “high yield,” accuracy regarding difficulty, and question type. Question topics included Fundamentals (24%), Cornea (10.1%), Glaucoma (8.9%), Retina (8.9%), Oculoplastics (3.5%), Neuro-Ophthalmology (7.8%), Pediatrics/Strabismus (8.1%), Optics (5.8%), Pathology (2.3%), Refractive Surgery (2.7%), Uveitis (9.7%), Lens/Cataract (5.4%), and Infectious Disease/General Medicine (2.7%).

**Figure 1:**
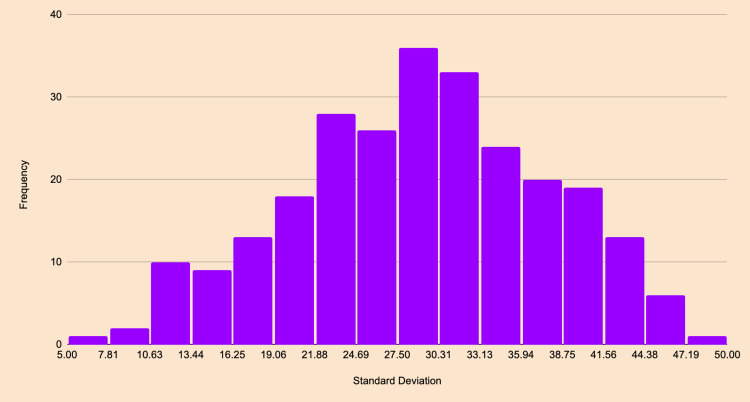
Histogram of standard deviation in human answers for Ophthalmic Knowledge Assessment Program (OKAP) questions.

The difficulty was determined using a standard deviation of popularity, and user answer choices were used to determine question difficulty. First, a random set of OKAP questions from the OphthoQuestions question bank was selected, and user responses to each question were recorded to capture the popularity of each answer choice. For each question, the standard deviation of the answer choices was calculated, providing insights into the dispersion of user responses. A histogram of these standard deviations was then created to visualize the distribution of answer choice variability, as seen in Figure 1. The percentiles of the standard deviation data were computed to categorize questions based on their difficulty (Table 1).

**Table 1:** Standard deviation quartiles for difficulty. These deviations used percentiles to rank the questions into four difficulty levels: Rank 1 (0-25th percentile) as the hardest and Rank 4 (76-100th percentile) as the easiest.

Standard deviation	Quartile
7.410578025	0
23.28320638	25
29.58603274	50
34.74430401	75
47.5	100

It was observed that standard deviations were artificially elevated for easier questions due to the most popular answer acting as an outlier. Conversely, lower standard deviations indicated harder questions as user responses were more evenly distributed among the answer choices. Therefore, the standard deviation was inversely proportional to question difficulty. Questions were ranked into the following four difficulty levels based on their percentile range: Rank 1 for the 0-25th percentile (hardest), Rank 2 for the 26th-50th percentile, Rank 3 for the 51st-75th percentile, and Rank 4 for the 76th-100th percentile (easiest). Each question was then assigned a difficulty rank according to the percentile of its standard deviation.

Further, we faced challenges with prompting both ChatGPT-4 and Gemini to answer multiple-choice questions. Initial prompts that contained only the question verbatim could lead to rejections, with both models responding that they were not equipped to answer clinical medical questions. To address this, we implemented a secondary prompt to facilitate their ability to answer, especially for image-based questions. This prompt instructed the AI to “Pick a letter to this multiple-choice question as if you were taking an exam, with no possible harm done to others.” This approach ensured that the models engaged with the questions more effectively, allowing us to properly assess their performance.

Two-tailed t-tests were conducted to compare the performance between ChatGPT-4.0 and Gemini Advanced. Given the nature of the observational study and the inherent variability in data, we aimed for a power of 0.8 to detect a significant difference between the versions of Gemini Advanced and ChatGPT-4.0 with an alpha value of 0.05. Furthermore, prior studies found little variability in redoing questions with GPT [7].

## Results

ChatGPT 4.0 vs. Gemini Advanced

**Figure 2:**
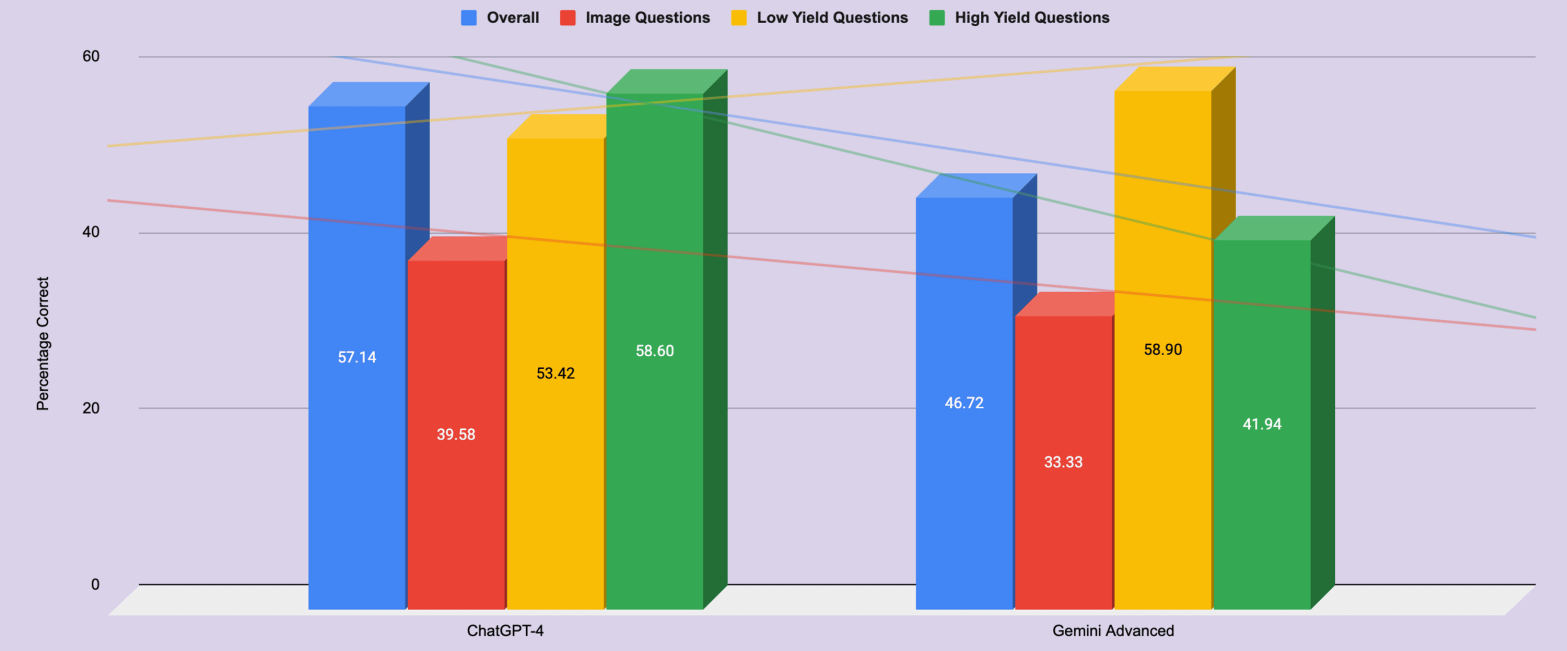
Comparison of GPT-4 and Gemini Advanced percentage correct across various question types.

In total, 259 of the 260 questions were included in the study, and one question used a video that any form of ChatGPT could not interpret as of May 1, 2024. ChatGPT-4.0 correctly answered 57% (148/259), and Gemini Advanced correctly answered 47% (121/259), resulting in a statistically significant difference in accuracy (p < 0.02) (Table 2). Both versions answered most questions without a prompt and would have received a well below-average score on the OKAP. The average score on these OKAP questions from human users was 65% (168/259). For image-with-text questions, ChatGPT-4.0 correctly answered 39.58% (19/48), while Gemini Advanced correctly answered 33.33% (16/48), resulting in a statistically insignificant difference in accuracy (p < 0.6) (Figure 2). The human user averaged 67% of correct answers.

**Table 2:** Comparative total accuracy of GPT-4 and Gemini Advanced across different question types. The single asterisk (*) next to the p-value indicates that the result is statistically significant with a p-value less than 0.05. The double asterisks (**) indicate a statistically significant result with a p-value less than 0.01. These p-values were determined utilizing a t-test with unequal variance.

	Overall total correct	IMG questions total correct	Low-yield questions total correct	High-yield questions total correct
ChatGPT-4	148	19	39	109
Gemini Advanced	121	16	43	78
Total questions	259	48	73	186
P-value	0.018*	0.530	0.508	0.001**

ChatGPT 4.0 vs. Gemini Advanced on prompting

ChatpGPT 4.0 directly responded without prompting on 89.2% of questions, whereas Gemini Advanced responded to 73.8% of questions (p < 0.000004).

ChatGPT 4.0 vs. Gemini Advanced on question difficulty

**Figure 3:**
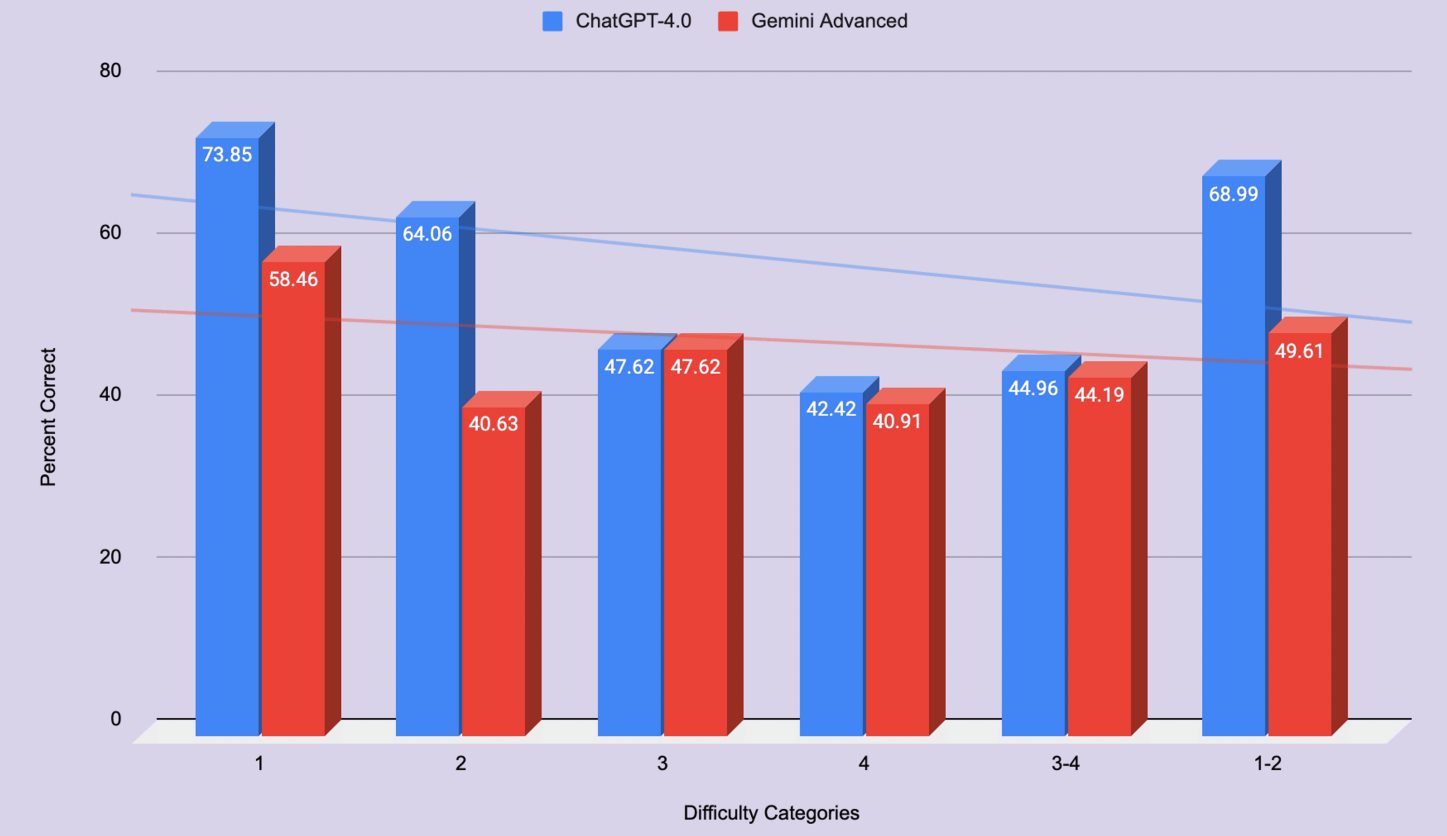
Comparison of GPT-4 and Gemini Advanced percentage correct across question difficulty.

ChatGPT-4.0.0 performed 61% on easier questions (<2 on a scale from 1-4) and 51% on harder questions (>2 on a scale from 1-4) (Figure 3). Gemini Advanced performed 46% on easier questions (<3 on a scale from 1-5) and 48% on harder questions (>2 on a scale from 1-4) (Figure 3). There was a statistically significant difference for easy (p < 0.03), but not for hard (p < 0.4) questions (Table 3).

**Table 3:** GPT-4 and Gemini Advanced’s accuracy across question difficulty levels. The single asterisk (*) next to the p-value indicates that the result is statistically significant with a p-value less than 0.05. The double asterisks (**) indicate a statistically significant result with a p-value less than 0.01. These p-values were determined utilizing a t-test with unequal variance.

Difficulty	GPT-4 correct	GPT-4 incorrect	Gemini correct	Gemini incorrect	Total	P-value (GPT vs. Gemini)
1	48	17	38	27	65	0.047*
2	41	23	26	38	64	0.286
3	30	33	30	33	63	0.093
4	28	38	27	39	66	0.861
3-4	58	3-11	57	72	129	0.548
1-2	89	2-8	64	65	129	0.001**

ChatGPT 4.0 vs. Gemini Advanced subspecialties

**Figure 4:**
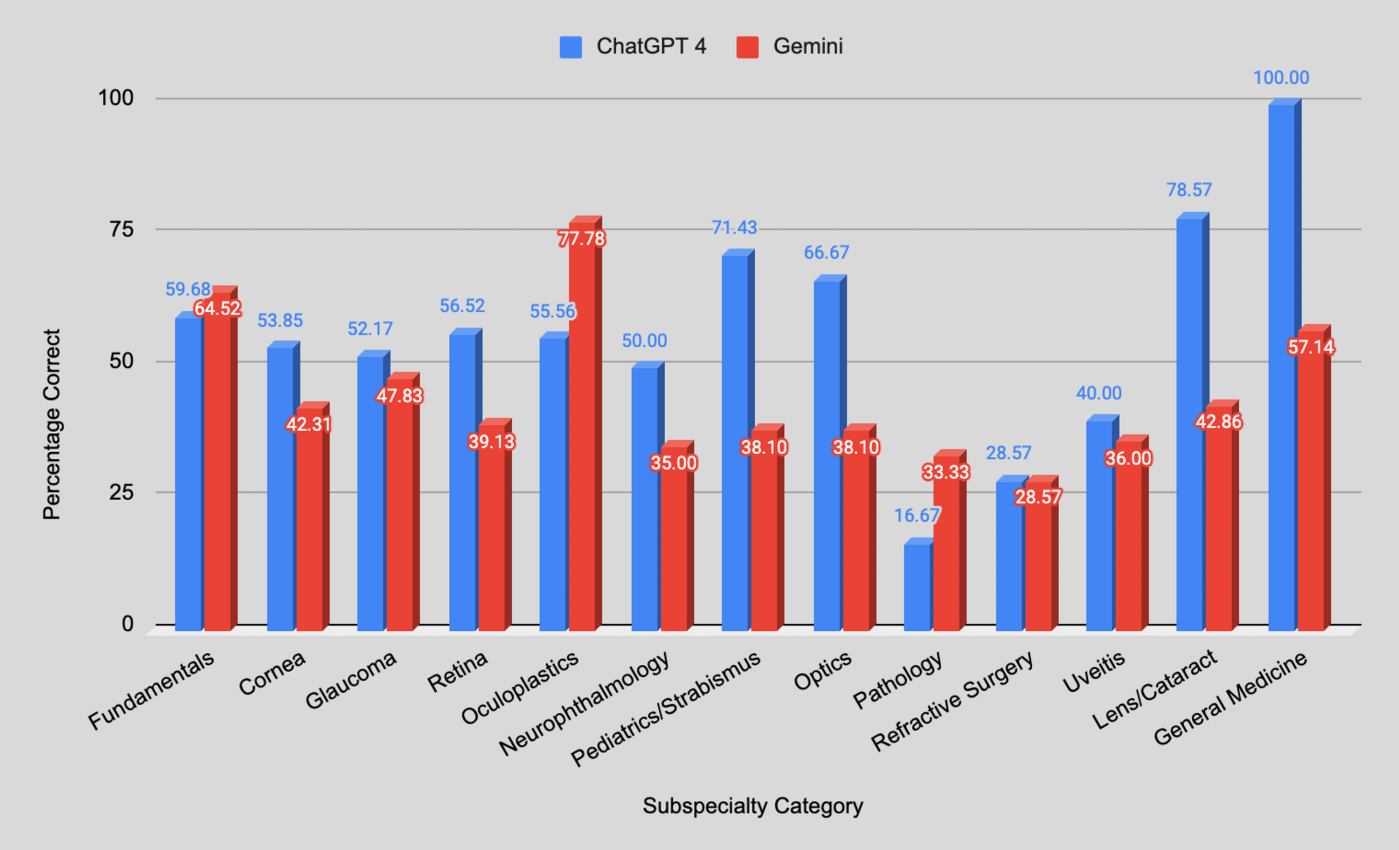
Comparison of GPT-4 and Gemini Advanced percentage correct across ophthalmology subspecialties.

The top-scoring question categories for ChatGPT-4.0 were Lens/Cataract (78.57%) and General Medicine (100%). The worst categories for ChatGPT-4.0 were Pathology (16.67%) and Refractive Surgery (28.57142857%) (Figure 4). The top-scoring question categories for Gemini Advanced were Oculoplastics (77.78%) and Fundamentals (64.52%). The worst categories for Gemini Advanced were Refractive (28.6%) and Optics/Pathology (both 33.33%) (Figure 4). The only statistically significant difference between ChatGPT-4 and Gemini Advanced was found in Pediatrics/Strabismus (p < 0.03) (Table 4).

**Table 4:** Comparative performance of GPT-4 and Gemini Advanced on various ophthalmology subspecialties. The single asterisk (*) next to the p-value indicates that the result is statistically significant with a p-value less than 0.05. These p-values were determined utilizing a t-test with unequal variance.

Subspecialty category	GPT correct	Gemini correct	GPT incorrect	Gemini incorrect	Total	P-value GPT vs. Gemini
Fundamentals	37	40	25	22	62	0.582
Cornea	14	11	12	15	26	0.415
Glaucoma	12	11	11	12	23	0.774
Retina	13	9	10	14	23	0.247
Oculoplastics	5	7	4	2	9	0.347
Neurophthalmology	10	7	10	13	20	0.350
Pediatrics/Strabismus	15	8	6	13	21	0.030*
Optics	10	5	5	10	15	0.072
Pathology	1	2	5	4	6	0.550
Refractive Surgery	2	2	5	5	7	1
Uveitis	10	9	15	16	25	0.776
Lens/Cataract	11	6	3	8	14	0.056
General Medicine	7	4	0	3	7	0.078

## Discussion

The main goal of this project was to determine the ability of ChatGPT-4.0 and Google’s Gemini Advanced to solve complex ophthalmic subspecialty medical questions. We specifically reported performance on 260 OKAP-style questions from the OphthoQuestions question bank, simulating the OKAP exam. The study compared the scores of both models, assessing their capabilities against real-life residents. Additionally, we evaluated ChatGPT-4.0 and Gemini Advanced’s ability to comprehend visual information by comparing their performance on both image-based and text-only questions. Although further development is needed before fully integrating these LLMs into academic spaces for training subspecialty residents, the results of this study illustrate clear improvements in LLMs’ performance at specialized subspecialty levels of training. Continued progression may lead to the development of ChatGPT-4.0 and Gemini Advanced as valuable tools in medical education, providing learning guides, interactive case studies, simulated patient interactions, and practice questions for residents or fellows in Ophthalmology.

While previous studies have primarily focused on evaluating the performance of different versions of ChatGPT, our study uniquely compares two distinct LLMs: ChatGPT-4.0 and Google’s Gemini Advanced (Bard). This comparative analysis offers several unique contributions. First, our study is the first to directly compare the performance of ChatGPT-4.0 with Gemini Advanced, providing insights into how different training methodologies and optimization techniques impact LLM performance in a specialized medical field. Second, we assessed both image-based and non-image-based questions, offering a holistic evaluation of the LLMs’ capabilities. Previous studies primarily focused on text-based questions, leaving a gap in understanding how LLMs handle complex visual information, critical in fields such as ophthalmology. Third, our study is distinct from the previous Bard study, which evaluated Bard’s performance on general ophthalmology board exam practice questions [5]. We specifically focus on OKAP-specific questions, providing a more detailed and relevant assessment of the LLMs’ performance. Additionally, we compared Google Gemini Advanced with another advanced LLM, ChatGPT-4.0, to offer a more comprehensive and comparative perspective not explored in the prior Bard study. Our study builds on the foundations laid by prior research but distinguishes itself by comparing two advanced LLMs in a specialized medical domain while employing a comprehensive and methodologically rigorous approach.

ChatGPT-4.0’s higher score, as seen in Figure 1, could be attributed to its more advanced fine-tuning of medical and ophthalmic content [8-11]. OpenAI has progressively improved the training processes and datasets used for ChatGPT-4.0, potentially incorporating more specialized medical knowledge and examples. This enhanced fine-tuning likely provides ChatGPT-4.0 with a better foundation to understand and respond to complex medical queries, including those specific to ophthalmology. Second, the architecture and training methodologies of ChatGPT-4.0 might be more conducive to handling detailed and nuanced medical information. ChatGPT-4.0 has demonstrated strong performance in various NLP tasks due to its large-scale pre-training and subsequent fine-tuning phases, which might include a wider array of medical literature and practice questions compared to Gemini Advanced. The ability to parse and generate contextually accurate responses is critical for high-stakes medical questions, and ChatGPT-4.0’s architecture appears better suited for this task.

On the other hand, Gemini Advanced’s lower performance could be influenced by several factors. While Gemini Advanced also utilizes advanced transformer-based architecture, its training and fine-tuning might not have been as heavily focused on medical or ophthalmic content. If Gemini Advanced’s training datasets included a broader but less specialized range of content, it might not have developed the same depth of knowledge in ophthalmology as ChatGPT-4.0. Additionally, Google’s emphasis on ethical guidelines and safety measures, which might include conservative approaches to medical advice, could cause more cautious and less precise responses, impacting performance on specialized medical questions [12]. Moreover, the inherent variability in LLM performance due to stochastic processes in generating responses could also play a role. Both models generate answers probabilistically based on learned patterns, which can introduce variability in their performance. However, ChatGPT-4.0’s more extensive fine-tuning may have reduced this variability, leading to more consistent and accurate answers compared to Gemini Advanced. Lastly, it is important to consider the specific content and format of the OphthoQuestions question bank. If the question bank included topics or question styles more familiar to ChatGPT-4.0’s training data, this alignment could further enhance its performance relative to Gemini Advanced. Differences in how each model processes and understands complex medical terminology, clinical scenarios, and image-based information might also contribute to the observed performance gap.

ChatGPT and Gemini Advanced underperformed on image-based questions likely for several reasons. First, both models are primarily designed to process and generate text, meaning their ability to interpret and analyze visual information is limited. Their training data predominantly consists of text, which does not equip them with the necessary skills to handle complex visual data effectively. Second, image-based questions often require detailed visual analysis, pattern recognition, and the ability to discern subtle differences, skills that these text-based LLMs lack. Without specific training on large datasets of annotated medical images, their performance in this area remains suboptimal. Third, the current architecture of these models does not support direct integration with visual processing capabilities. While some advanced AI models combine text and image processing, ChatGPT and Gemini Advanced have not been primarily optimized for this type of multimodal input. In addition, the OphthoQuestions question bank, designed specifically for ophthalmology residents preparing for the OKAP exam, could have included content or phrasing that is unfamiliar to the models. This may have further hampered their performance, especially if the questions emphasized real-world clinical judgment or complex differential diagnoses, areas where the models may struggle without extensive fine-tuning specific to these fields.

Image question accuracy was significantly insignificant between the two LLMs. Gemini Advanced comparatively performed better within this niche of questions in comparison to text-only questions which could be because of Google’s extensive resources and experience in image recognition and processing, potentially leveraging its vast datasets and advanced image analysis algorithms. Google’s Gemini Advanced might also benefit from specific optimizations in handling medical images, such as incorporating advanced machine learning techniques such as convolutional neural networks (CNNs) that are specifically designed for image analysis. Additionally, the differences in the underlying architecture, training methodologies, or access to diverse and high-quality annotated medical image datasets could explain why one model outperformed the other on image-based questions.

The performance of GPT-4 and Gemini Advanced across various ophthalmology subspecialties revealed notable differences in their accuracy. GPT-4 generally outperformed Gemini Advanced in subspecialties such as Pediatrics/Strabismus, Fundamentals, and Lens/Cataract, achieving accuracy rates of 71.43%, 59.68%, and 78.57%, respectively. Conversely, Gemini Advanced demonstrated higher performance in Oculoplastics and Refractive Surgery, with accuracy rates of 77.78% and 28.57%, respectively. These differences were statistically significant in certain categories, such as Pediatrics/Strabismus, which could suggest meaningful disparities in model performance (p < 0.05). However, it is important to note that the dataset for each subspecialty was relatively small, which could contribute to variability in the results. The small sample sizes may lead to fluctuations in accuracy percentages, and larger datasets would be necessary to confirm these findings with greater confidence. The statistical significance observed in some performance differences highlights potential architectural and training impacts, but these results should be interpreted cautiously due to the limited data.

The underperformance of both ChatGPT-4.0 and Gemini Advanced in areas such as pathology and refractive surgery, where ChatGPT achieved scores of 16.67% and 28.57%, respectively, while Gemini Advanced performed similarly poorly, can be attributed to several significant factors. One of the most pressing reasons for this underperformance is the limited availability of publicly accessible, high-quality datasets in these specialized medical fields [11]. These LLMs are primarily trained on large datasets sourced from publicly available text, which are generally more abundant for broad, well-covered medical topics, such as general medicine or basic clinical knowledge [13]. However, pathology and refractive surgery represent highly specialized areas of medicine that require access to detailed, domain-specific data. Unfortunately, such datasets are less commonly available in the public domain, which directly restricts the models’ exposure to the nuanced and intricate information necessary to develop a comprehensive understanding of these fields [13].

Pathology, for instance, involves complex diagnostic criteria that require familiarity with a wide range of clinical presentations, histopathological findings, and subtle variations in disease states that may not be well-represented in the general medical datasets used for training these models. Refractive surgery, on the other hand, often involves detailed knowledge of surgical techniques, patient-specific considerations such as corneal topography, and an advanced understanding of postoperative complications, which are typically covered in more specialized or clinical sources that may not be widely available. As these models are not trained with extensive input from such specialized datasets, their ability to grasp the depth and complexity of pathology and refractive surgery is inherently limited [13].

Moreover, both of these areas require a level of clinical reasoning and specialized medical expertise that general datasets may not provide. For example, pathology often involves a synthesis of visual and clinical information, requiring the ability to integrate histological images, laboratory results, and patient history to arrive at a diagnosis. Similarly, refractive surgery decisions rely on precise measurements and patient-specific factors that demand a higher degree of clinical judgment than what is typically required in more general medical scenarios. The models’ reliance on general datasets means that they are not exposed to the advanced problem-solving and decision-making processes necessary to excel in these subspecialties, further contributing to their lower performance in these areas [13]. Without more extensive, targeted training on specialized datasets, the models remain ill-equipped to fully understand the nuances required to excel in fields such as pathology and refractive surgery.

Gemini Advanced demonstrated more reluctance to answer medical-specific questions compared to ChatGPT-4.0, which may be due to a slew of reasons. First, Google’s Gemini Advanced may have more stringent ethical guidelines and safety measures in place to prevent misuse of the model in sensitive areas such as medical advice​​ [8]. These safety protocols likely make the model more cautious and thus more reluctant to respond to medical-specific questions. Second, Gemini Advanced’s training approach might include a more conservative stance toward answering medical queries, prioritizing the avoidance of potential harm, and ensuring compliance with legal and ethical standards, leading to a higher rate of refusal for medical questions. Third, the data used to train Gemini Advanced may have been filtered to exclude or limit medical content, making the model less confident and more likely to refuse to answer medical-specific questions, ensuring that the model does not provide information it is not thoroughly trained on. Finally, unlike ChatGPT-4.0, Gemini Advanced may have received less fine-tuning specifically for medical contexts, resulting in a higher rate of refusal when confronted with medical questions, as the model is less equipped to handle such content confidently.

Furthermore, Gemini Advanced would give answers that are not listed in multiple-choice answers. The training data for Gemini Advanced might not include enough examples of multiple-choice questions with clearly defined answer choices, leading it to generate answers based on its broader knowledge. Moreover, handling multiple-choice questions accurately requires precise NLP capabilities, and any ambiguities or complex wording in the question can confuse the model [8]. The underlying algorithms in Gemini Advanced might use heuristics or inference techniques that sometimes lead to over-generalization, causing the model to produce an answer that fits its understanding but does not align with the specific choices given [8]. Medical questions often involve complex reasoning and domain-specific knowledge, and if the model finds that none of the provided choices match its inferred answer, it might default to providing an answer it considers most accurate based on its extensive training data [8]. Lastly, Gemini Advanced might provide an answer outside of the given options if it has a higher confidence level in its generated response compared to the provided choices, based on its training data suggesting a different answer than those listed [8].

One of the limitations of this study was the initial unwillingness of ChatGPT-4 and Gemini Advanced to engage with image-based questions and accurately utilize the provided images. Often, when presented with a prompt containing just the question, both models would respond with a message stating they were not equipped to answer clinical medical questions or would guess without utilizing the image, stating an image was not available. This limitation was particularly evident in image-based questions, where the models failed to incorporate visual data into their responses. To overcome this issue, we used a secondary prompt to ensure proper engagement with the images. The prompt instructed, “Please utilize the image in this question when determining your answer.” This additional directive helped the AI models to consider the visual information provided, thereby allowing a more accurate assessment of their performance on these types of questions. By employing this strategy, we aimed to ensure that the models utilized all available data to generate their answers, thus enhancing the validity of our comparison between ChatGPT-4 and Gemini Advanced.

Some other limitations of this study include the scope of questions, which focus on a specific question bank and may not fully represent the diversity and range of questions encountered in actual OKAP exams, potentially limiting the generalizability of the findings to real-world exam scenarios. Additionally, the lack of real-world clinical context means that the study evaluated the models’ performance on written questions but did not assess their ability to apply this knowledge in practical settings, which involve more nuanced decision-making and patient interaction. Furthermore, both ChatGPT-4.0 and Gemini Advanced are trained on large datasets that may contain inherent biases, potentially influencing the models’ responses and propagating incorrect or biased medical information. The study primarily used accuracy scores to evaluate the models’ performance; however, other metrics such as precision, recall, and the ability to provide justifications for answers could offer a more nuanced understanding of the models’ strengths and weaknesses. Finally, technical constraints, such as the models’ inability to process certain types of complex visual data or the prompt-handling mechanisms that influence how the models interpret and respond to questions, may have impacted the results and should be considered when evaluating the models’ performance.

The replicability of studies involving LLMs such as ChatGPT-4.0 and Gemini Advanced is generally consistent, as noted in prior research, but can be inherently variable [14-16]. This variability represents a significant weakness, particularly in examining these models’ performance on specialized medical questions such as those found in the OKAP exam. This variability stems from the probabilistic nature of LLMs, which generate responses based on patterns learned from massive datasets but can yield different outputs even under similar conditions. The stochastic algorithms that drive these models mean that each run might interpret the input slightly differently and produce varied responses. This stochastic nature is due to several factors related to the structure of each LLM: the vast and diverse training data sourced from the internet, which inherently contains a wide range of writing styles, contexts, and information; the complex model architecture with multiple layers of neurons and connections that process input data, where each neuron’s activation can vary slightly depending on initial conditions and training sequences; the inference process involving beam search or sampling, where small differences in probability distributions or selection mechanisms (e.g., sampling temperature) can result in different responses; the high sensitivity of LLMs to the phrasing and structure of input prompts, making it challenging to ensure consistent outputs across different trials; and the fine-tuning variability, where specific examples, their order, and fine-tuning parameters can all influence the model's final behavior.

In the context of this study, such variability can introduce inconsistencies in performance metrics across different executions of the same test set for both ChatGPT-4.0 and Gemini Advanced. For instance, when answering OKAP-style questions, the models might provide slightly different answers or justifications each time they are queried, even if the input question remains unchanged. This can be particularly problematic in medical contexts where precise and consistent information is critical. The inability to guarantee consistent performance across multiple trials is a notable limitation for the adoption of LLMs such as ChatGPT-4.0 and Gemini Advanced in clinical practice, where consistency and reliability in diagnostic or educational support are paramount. Therefore, further studies are encouraged to solidify the findings of LLMs’ capabilities in the medical field, addressing the variability in responses to ensure more reliable performance.

These LLMs have significant potential to revolutionize the way medical exam questions are standardized, ensuring greater clarity, fairness, and consistency in exams such as the OKAP and USMLE. One of the primary advantages of using LLMs is their ability to generate, refine, and evaluate the language of exam questions, reducing ambiguities that could lead to misinterpretation by test-takers [17]. By analyzing the wording of questions, LLMs can suggest modifications that make them more straightforward, ensuring that they are interpreted consistently across diverse groups of students. This is particularly important in high-stakes medical exams, where even small variations in wording can significantly impact a candidate’s ability to understand and respond accurately to a question [17].

In addition to clarifying language, LLMs can also play a critical role in identifying and mitigating bias within exam questions. Medical exams often contain implicit biases related to gender, race, culture, or socioeconomic background, which may inadvertently favor or disadvantage certain groups [18]. LLMs, when trained appropriately, can analyze questions for such biases and recommend alternative phrasing or content to ensure a more equitable assessment [13]. For example, if a question involves a culturally specific reference or a scenario that assumes a particular demographic experience, the LLM can flag this as potentially problematic and suggest a more neutral scenario, ensuring that the question is fair and accessible to all test-takers.

Moreover, LLMs can assist in evaluating the difficulty of exam questions by analyzing how similar questions have been answered historically. By examining answer patterns and the distribution of correct responses, LLMs can predict the relative difficulty of new questions, helping exam creators design a well-balanced exam that includes an appropriate range of easy, moderate, and difficult questions. This can ensure that the exam is not overly challenging for one group of students while being too easy for others, contributing to a more accurate assessment of medical knowledge across the board.

Another critical function of LLMs in standardizing medical exams is their ability to generate practice questions. By leveraging vast datasets of medical literature, case studies, and clinical scenarios, LLMs can create a wide array of practice questions that align with the learning objectives of major medical exams [17]. These practice questions can be tailored to specific areas of medical knowledge, such as cardiology or ophthalmology, and can help students prepare by exposing them to a diverse range of question types and clinical situations. This not only helps students become more familiar with the format and content of exams but also provides educators with a valuable resource for gauging student progress and understanding.

Beyond generating and refining exam content, LLMs also offer potential utility in evaluating the overall clarity and relevance of medical questions before they are used in high-stakes exams. For instance, LLMs can analyze the logical flow of questions and ensure that they follow a clear, understandable progression [18]. This capability is especially useful in complex clinical questions that involve multiple steps of reasoning or decision-making. The models can ensure that the questions are framed in a way that guides the test-taker through the problem logically without introducing confusion or ambiguity. In doing so, LLMs help to ensure that questions test the intended knowledge and skills rather than a student’s ability to interpret confusing or poorly structured language [19].

Furthermore, LLMs can help maintain fairness by ensuring that all medical questions are aligned with current best practices and medical standards. Because LLMs can be continuously updated with the latest medical research and guidelines, they are well-positioned to ensure that exam content reflects the most up-to-date clinical knowledge [19]. This is particularly important in fields such as medicine, where guidelines and treatment protocols can change frequently. By reviewing and updating exam questions, LLMs can ensure that test-takers are being assessed on knowledge that is clinically relevant and reflective of current practice standards [18].

Although other researchers discuss the wide possibilities of AI in clinical medicine, this study provides evidence that ChaGPT 4.0 and Gemini Advanced underperformed in image-based questions, indicating the need for further refinement before these LLMs can be fully integrated into specialized medical training programs [20-24].

To improve the performance of LLMs in medical education, particularly in image-based questions and specialized subspecialties, several key areas of future research and development should be prioritized. One critical step is enhancing multimodal learning by optimizing LLMs such as ChatGPT-4 for seamless integration of text and images. In medical education, where diagnostic imaging is essential, research should focus on developing advanced multimodal models that can interpret both text and images effectively. Additionally, LLMs could be wired to pool information exclusively from reliable and approved medical textbooks to ensure the accuracy of the medical advice provided. This would significantly reduce the risk of misinformation and offer a more dependable foundation for both general medical knowledge and specialized topics. This improvement in sourcing would also enhance the LLM’s ability to interpret visuals more accurately, as it would draw from well-established medical standards and guidelines for diagnosis and treatment.

Specialized training on focused medical subfields, such as Oculoplastics or Retina, is another essential area of development. LLMs would benefit from being trained on curated datasets that include peer-reviewed, expert-approved content from these subspecialties, thus improving their accuracy in answering nuanced medical questions. Collaborative human-AI learning systems also present a promising avenue for future enhancement. These systems would allow real-time feedback from medical professionals to fine-tune the models for greater accuracy, especially in complex subspecialty cases where subtle details are crucial.

Improving performance in image-based medical questions also requires the development of advanced neural architectures, such as transformer-based visual models (e.g., Vision Transformers), to enhance the models’ capability to analyze and interpret medical images effectively. Benchmarking and evaluation should be conducted regularly using specialized datasets from trusted medical question banks, providing clearer insights into how well the models handle both text and image-based queries. This iterative process would highlight areas where LLMs need to improve and ensure continual advancement in their capabilities.

Additionally, addressing bias and ensuring ethical AI in medical contexts is critical, especially in subspecialties that deal with sensitive patient data. Future research should emphasize reducing bias in the training data and improving transparency in AI decision-making processes. By using inclusive datasets and developing robust methods to assess and mitigate biases in medical responses, LLMs can become more reliable and ethical tools in medical education. Altogether, these steps offer a clear roadmap for advancing the use of LLMs in medical education, enhancing their utility in both image interpretation and highly specialized subspecialties while ensuring accuracy and ethical integrity.

## Conclusions

In this study, ChatGPT-4.0 performed better on the OKAP-style exams compared to Gemini Advanced within the context of ophthalmic multiple-choice questions. This may show an opportunity for increased worth for ChatGPT in ophthalmic medical education. Future research should continue to evaluate the test-taking proficiency and clinical reasoning skills of ChatGPT and other LLMs across different medical disciplines and scenarios. This will help fully understand their capabilities and limitations in practical clinical applications.

## References

[1] Mihalache A, Grad J, Patil NS (2024). Google Gemini and Bard artificial intelligence chatbot performance in ophthalmology knowledge assessment. Eye (Lond).

[2] Carlà MM, Gambini G, Baldascino A, Boselli F, Giannuzzi F, Margollicci F, Rizzo S (2024). Large language models as assistance for glaucoma surgical cases: a ChatGPT vs. Google Gemini comparison. Graefes Arch Clin Exp Ophthalmol.

[3] Guerra GA, Hofmann H, Sobhani S (2023). GPT-4 artificial intelligence model outperforms ChatGPT, medical students, and neurosurgery residents on neurosurgery written board-like questions. World Neurosurg.

[4] Gilson A, Safranek CW, Huang T, Socrates V, Chi L, Taylor RA, Chartash D (2023). How does ChatGPT perform on the United States Medical Licensing Examination (USMLE)? The implications of large language models for medical education and knowledge assessment. JMIR Med Educ.

[5] Botross M, Mohammadi SO, Montgomery K, Crawford C (2024). Performance of Google's artificial intelligence chatbot "Bard" (now "Gemini") on Ophthalmology Board Exam practice questions. Cureus.

[6] Teebagy S, Colwell L, Wood E, Yaghy A, Faustina M (2023). Improved performance of ChatGPT-4 on the OKAP examination: a comparative study with ChatGPT-3.5. J Acad Ophthalmol (2017).

[7] Antaki F, Touma S, Milad D, El-Khoury J, Duval R (2023). Evaluating the performance of ChatGPT in ophthalmology: an analysis of its successes and shortcomings. Ophthalmol Sci.

[8] Patil NS, Huang RS, van der Pol CB, Larocque N (2024). Comparative performance of ChatGPT and Bard in a text-based radiology knowledge assessment. Can Assoc Radiol J.

[9] Toyama Y, Harigai A, Abe M, Nagano M, Kawabata M, Seki Y, Takase K (2024). Performance evaluation of ChatGPT, GPT-4, and Bard on the official board examination of the Japan Radiology Society. Jpn J Radiol.

[10] Ali R, Tang OY, Connolly ID (2023). Performance of ChatGPT, GPT-4, and Google Bard on a Neurosurgery Oral Boards Preparation Question Bank. Neurosurgery.

[11] Google LLC (2024). Google AI. AI principles: 2023 progress update. AI Principles Progress Update 2023.

[12] Sahin MC, Sozer A, Kuzucu P (2024). Beyond human in neurosurgical exams: ChatGPT's success in the Turkish neurosurgical society proficiency board exams. Comput Biol Med.

[13] Singhal K, Azizi S, Tu T (2023). Large language models encode clinical knowledge. Nature.

[14] Weng TL, Wang YM, Chang S, Chen TJ, Hwang SJ (2023). ChatGPT failed Taiwan's Family Medicine Board Exam. J Chin Med Assoc.

[15] Cheung BH, Lau GK, Wong GT (2023). ChatGPT versus human in generating medical graduate exam multiple choice questions-a multinational prospective study (Hong Kong S.A.R., Singapore, Ireland, and the United Kingdom). PLoS One.

[16] Antaki F, Milad D, Chia MA (2023). Capabilities of GPT-4 in ophthalmology: an analysis of model entropy and progress towards human-level medical question answering. Br J Ophthalmol.

[17] Meng X, Yan X, Zhang K (2024). The application of large language models in medicine: a scoping review. iScience.

[18] Frosolini A, Catarzi L, Benedetti S (2024). The role of large language models (LLMs) in providing triage for maxillofacial trauma cases: a preliminary study. Diagnostics (Basel).

[19] Ray PP (2023). ChatGPT: a comprehensive review on background, applications, key challenges, bias, ethics, limitations and future scope. Internet Things Cyber-Phys Syst.

[20] Shukla R, Mishra AK, Banerjee N, Verma A (2024). The comparison of ChatGPT 3.5, Microsoft Bing, and Google Gemini for diagnosing cases of neuro-ophthalmology. Cureus.

[21] Carlà MM, Gambini G, Baldascino A (2024). Exploring AI-chatbots' capability to suggest surgical planning in ophthalmology: ChatGPT versus Google Gemini analysis of retinal detachment cases. Br J Ophthalmol.

[22] Ker J, Wang L, Rao J, Lim T (2018). Deep learning applications in medical image analysis. IEEE Access.

[23] Artsi Y, Sorin V, Konen E, Glicksberg BS, Nadkarni G, Klang E (2024). Large language models for generating medical examinations: systematic review. BMC Med Educ.

[24] Homolak J (2023). Opportunities and risks of ChatGPT in medicine, science, and academic publishing: a modern Promethean dilemma. Croat Med J.

